# A Year at the Forefront of Hydrostat Motion

**DOI:** 10.1242/bio.059834

**Published:** 2023-08-10

**Authors:** Andrew K. Schulz, Nikole Schneider, Margaret Zhang, Krishma Singal

**Affiliations:** ^1^ School of Mechanical Engineering, Georgia Institute of Technology, Atlanta, GA 30332, USA; ^2^ Department of Biology, University of South Dakota, Vermillion, SD 57069, USA; ^3^ School of Physics, Georgia Institute of Technology, Atlanta, GA 30332, USA

**Keywords:** Muscular hydrostat, Hydro-skeleton, Morphology, Biomechanics, Bio-inspiration, Muscle, Knitting

## Abstract

Currently, in the field of interdisciplinary work in biology, there has been a significant push by the soft robotic community to understand the motion and maneuverability of hydrostats. This Review seeks to expand the muscular hydrostat hypothesis toward new structures, including plants, and introduce innovative techniques to the hydrostat community on new modeling, simulating, mimicking, and observing hydrostat motion methods. These methods range from ideas of kirigami, origami, and knitting for mimic creation to utilizing reinforcement learning for control of bio-inspired soft robotic systems. It is now being understood through modeling that different mechanisms can inhibit traditional hydrostat motion, such as skin, nostrils, or sheathed layered muscle walls. The impact of this Review will highlight these mechanisms, including asymmetries, and discuss the critical next steps toward understanding their motion and how species with hydrostat structures control such complex motions, highlighting work from January 2022 to December 2022.

## Teaser

**Summary:** A review of the new innovations, technology, and hypothesis surrounding the structures, functions, and mechanisms of hydrostats.

## Introduction

The complexities of muscle are spread across several different biological magnitudes, from the micron scale of muscle neural transmitters to the macro scale of organized complex muscle groups that vary throughout the biological world. Muscles traditionally produce controlled movement through a combination of activity in joints, cartilage, bones, and skin; however, the exception of this traditional system is hydrostat motion. Hydrostats are described as systems in the anatomy of wildlife that provide movement but lack the typical system of skeletal support. These muscular organs maneuver like a fluid, maintaining volume conservation during complex tasks, such as obstacle maneuvering (Kier and Smith, 1985). The most common hydrostat categories include muscular hydrostats, such as tongues, tentacles, and trunks, and the hydroskeleton (aka, hydrostatic skeleton), which can be seen in starfish (Kier, 2012). These hydrostats have complex maneuverability as no rigid structures provide physical means to describe their degrees of freedom. Therefore, they have been termed infinite degrees of freedom (iDOF) systems (Zullo et al., 2022).

The diversity of hydrostat structures is further seen in the variety of compounds and mechanisms that give them these hydrostat abilities – in muscular hydrostats; we see the activation and contraction of muscle fibers leading to dexterous manipulation in the form of elongation, shortening, bending, and torsion (Kier and Smith, 1985). These fibers can be arranged in bundles of muscle units or laid out in sheets, and muscle ultrastructure can be modified to allow for extreme movement, such as ballistic tongue projection to capture prey items from a distance without risk of injury to the muscle (Wainwright and Bennett, 1992; de Groot and van Leeuwen, 2004; Anderson, 2016). The traditional hydrostat structure is seen as a fluid actuator in an appendage accomplishing complex movements while obeying volume conservation during deformation (Kier, 1992; Kier and Smith, 1985). We introduce a similar definition that breaks a hydrostat into three primary components similar to the bio-inspiration framework: structure, function, and mechanism (SFM).

Kier's definition of hydrostats and hydroskeletons allows for new hydrostats to fulfill this framework as we look into new appendages with fluid actuators that obey the conservation of volume, similar to Kier's proposal in 1985. This actuation diversity allows for new bio-inspiration types of soft robotics and soft structures (Trivedi et al., 2008) based on muscular hydrostats and hydroskeletons. Bio-inspired design has steadily progressed over the last century to create more realistic muscle mimics for various applications, including bio-inspired robotics. These hydrostat-inspired robotics are all working to find a suitable bio-inspired muscle through different actuators, and we have seen significant progress from the original mechanically rigid bio-inspired robots from the early 2000s.

**Fig. 1.**
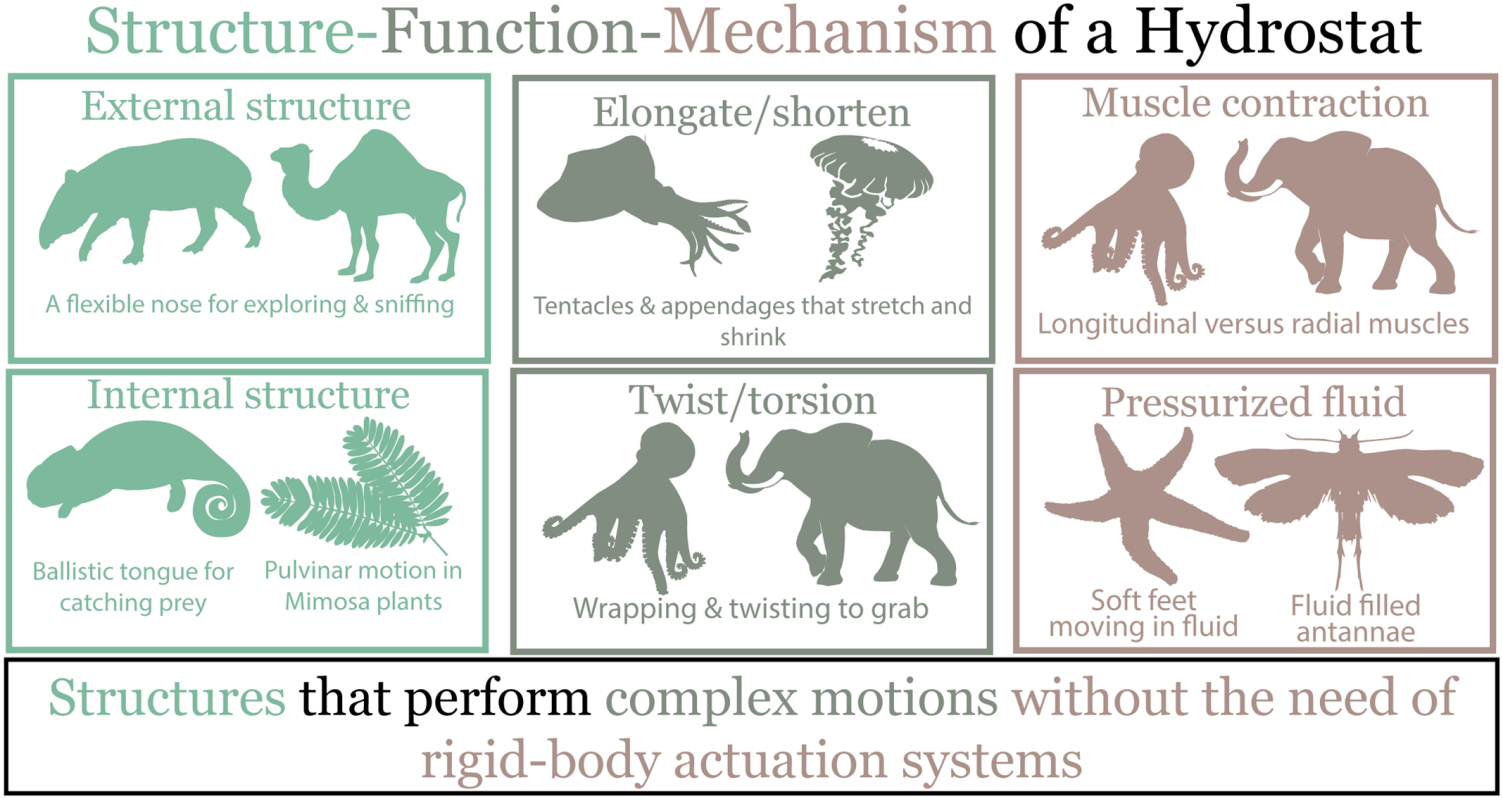
**The biodiversity of hydrostats is shown through their difference in structures, including tapir and camel proboscis-like noses, the tongues of a chameleon or cat, and the pulvinar motion in Mimosa plants.** Additionally, these hydrostats have complex motion capabilities or functions, including the tentacles of a squid, jellyfish, octopi, or elephant trunk. Finally, these motions are accomplished without needing a rigid-body actuation system, so the elephant trunk and octopus tentacle have no bones or joints, and the hydroskeletons of a moth proboscis and starfish use fluid pressure.

As we venture beyond the muscle and look to hydroskeletons, the bio-diversity of these structures is evident, ranging from hydroskeletons helping animals like starfish loco-mote or allowing movement in the armored plates of a scorpion (Yekutieli et al., 2009; Rosslenbroich, 2014). We have created an updated hydrostats framework using the structure-function-mechanism components normally discussed in bio-inspired or biomimicry applications (Zhang et al., 2022). We propose hydrostats are structures (structure) that perform complex motions (function) without the need for rigid-body actuation systems (a mechanism) (Fig. 1). The complexities of these hydrostats are the unifying factor of this field, and recently, new techniques and mechanisms have allowed researchers to understand or even mimic hydrostat functions, structures, or mechanisms. In this piece, we discussed the current state of hydrostat research in 2022 and the prospects for the future of hydrostat research expanding onto new types of hydrostat structures that accomplish complex motion through unique actuators.

## A Year at the Forefront of Hydro-static Motion

### Discoveries

The field of hydrostat research has rapidly grown over the past few years. The traditional hydrostat hypothesis of muscle movement in animals obeying conservation of volume is being expanded with recent discoveries of novel mechanisms. Several mammals have been found to have nose structures that fit within the bounds of a hydrostat. These nose structures can drastically differ based on the animal's environment. For example, the camel nose is different than other ruminant species. It is specialized as a vestibular proboscis, with adaptive features for dusty habitats, allowing for movement without the use of skeletal support (Latifi et al., 2022). The framework of structure, function, and mechanism of hydrostats discussed in the introduction has led to additional discoveries of each of these hydrostat themes.

Understanding plants and their connection to hydrostats through botanical biomechanics and physiology has continued to grow as a research area in the last few years. For decades botanists have studied the pulvinar motion in *Mimosa* through different types of imaging and study, such as nuclear magnetic resonance (NMR) imaging (Tamiya et al., 1988). Recently, the understanding of the structure, function, and mechanism of pulvinar motion in *Mimosa* has been extended through morphological and biomechanical experimentation (Sleboda et al., 2022). This work challenges the idea of hydrostats only applying to animals, expanding the hydrostat hypothesis to the field of botany. Pulvinar movement in *Mimosa* is possible with the pulvinus, which acts as a flexible joint between the stem and leaf stalk. Therefore, this structure has no rigid-body actuation system but is connected to relatively rigid plant members (the stem and the leaf stalk) (Volkov et al., 2010). This is similar to the elephant trunk connected to a boney skull, the tapir and camel nose to the skull, and so on. The comparative connection of plants and animal movement in this constrained actuation system is an area of future study.

**Fig. 2.**
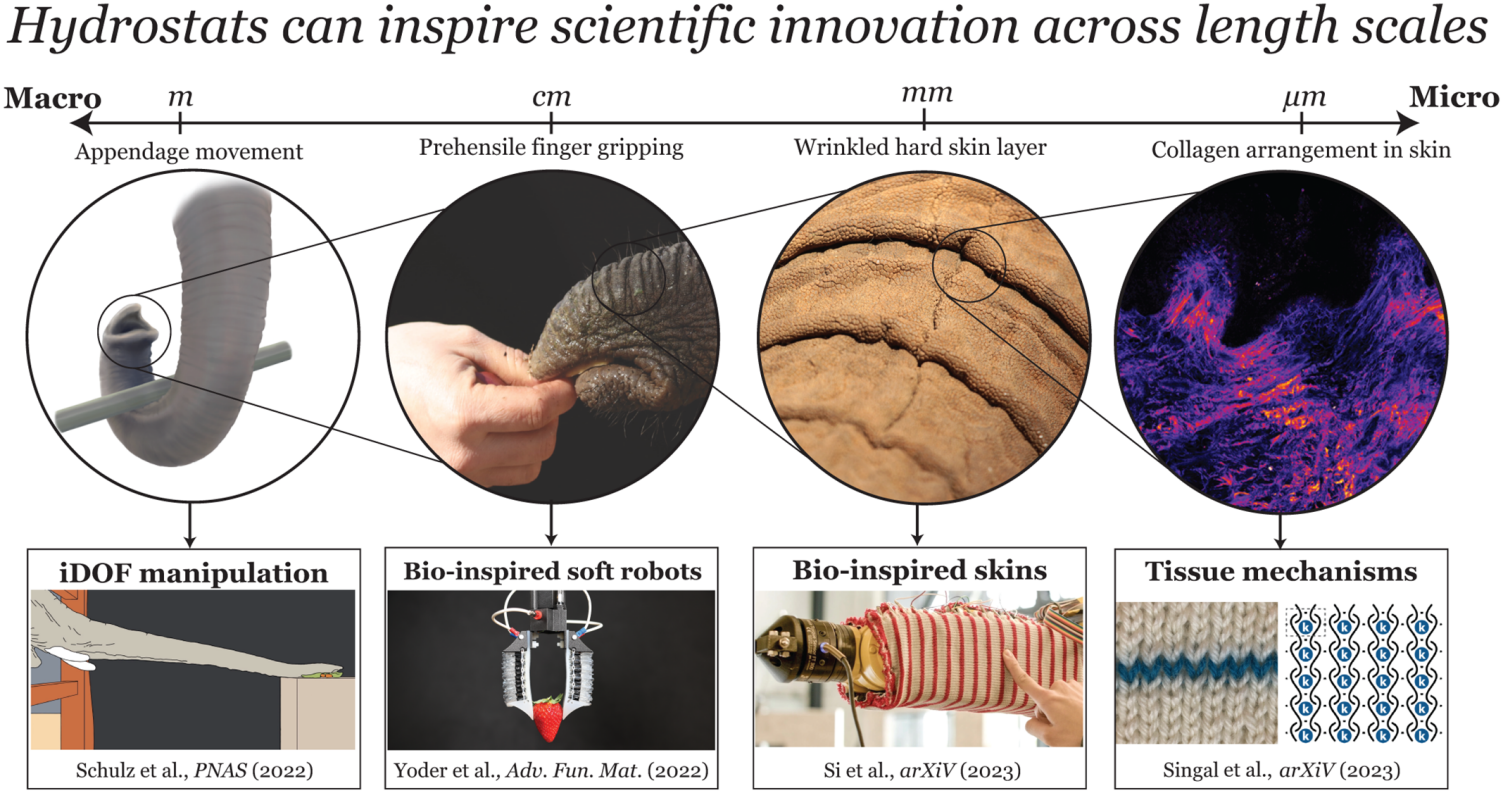
**Bio-inspiration and understanding across scales of a hydrostat ranging from appendage movements at the macro (m) scale helping investigate manipulation of infinite degree of freedom (iDOF) systems (****Schulz et al., 2022b****)**. At the cm scale, we see an elephant gripping with their two prehensile fingers to grab objects softly with different mechanisms to regulate force, with inspired grippers to have soft grasping ability (Yoder et al., 2022). At the mm scale, elephants have tough, wrinkled skin that is folded, and the ability of tough skin has inspired skins that cover robotics made of items such as knitting (Si et al., 2023 preprint). Finally, at the micro-scale (µm), we see collagen arrangement in the structural makeup of the dermal layer of skin has fiber-to-fiber interaction and can be understood using physics modeling of yarn-to-yarn interactions directing knitted fabric behavior (Singal et al., 2023 preprint).

The organization of movement from muscles can be viewed at various scales, where each level of the movement hierarchy has different types of activation (Fig. 2). Muscular control is an ever-expanding idea. Using a framework of physical models with experimental and biological testing, the activation control of plants can be driven using osmotically pressurized pulvini. These pulvini act as actuators in the plant stem allowing for complex movement to leverage varying kinematics of movement, similar to how a hydrostat organ operates (Sleboda et al., 2022). Additionally, we have seen specific discoveries in the past year in understanding some common muscular hydrostats, including tongues, trunks, and tentacles. Elephant trunks are elongated proboscis that are filled with muscles. When elongating, there is non-homogeneity during a stretch that is not from the muscle but from asymmetries in the skin properties (Schulz et al., 2022b) (Fig. 2). The dorsal skin is much stretchier than the ventral side, allowing the folded skin on the top to flatten for long elongation cycles. The ventral skin is stiff and has wrinkles throughout that the elephant utilizes for gripping objects. When lifting heavier barbells, they engage more wrinkles, allowing the added surface area for gripping with the stiff ventral skin (Schulz et al., 2023b). Octopuses also show morphological specialization of their muscles along the length of their arms to assist with control of arm motion (Di Clemente et al., 2021; Zullo et al., 2022). Additionally, asymmetries are seen in the tongues of mammals and reptiles, with the basic arrangement varying based on the feeding patterns and diet of the species (Ishaya et al., 2022). One of the underlying and unanswered questions of hydrostats is how they control these soft-bodied structures. Skeletal elements do not constrain them, so the arrangement of muscles in hydrostat structures is essential for movement and coordination. It has been found that elephants utilize a large set of facial control neurons to perceive motion and relative trunk location, with many concentrated at the tip (Kaufmann et al., 2022). These new discoveries are working on expanding the field of hydrostat motion beyond just the mechanics and closing the loop on the sensory structures that allow these complex movements to be accomplished.

### Technological innovations

A challenge in researching hydrostats is bringing these biological structures to the lab. How can we test them in a controlled environment? These structures are complex as they live in three-dimensional (3D) movement with infinite freedom, complicating the understanding of their muscular modality. Using motion tracking and movement structures, we can expand the ideas of two-dimensional (2D) hydrostat research to 3D (Feilich et al., 2021). Hydrostats are composed of muscles and muscle groups that can change in their arrangement from the tip to the base (Schulz et al., 2022b). There have been many innovations in simulation and theoretical investigations into modeling hydrostats and similar structures (Flash and Zullo, 2023; Wang et al., 2023; Sleboda et al., 2019). This includes understanding these hydrostats' geometry and composition through filament modeling (Kaczmarski et al., 2022). The idea of filaments and modeling them and their individual arrangement can generate vastly different mechanical responses as biological arrangements at multiple scales can generate complex individual interactions throughout living systems; an example of such is entanglement (Day et al., 2023). In physics, the study of entanglements affecting macro behavior is complex yet expanding. We understand that entanglement can lead to non-linear stress-strain response behavior (Bozdag et al., 2023). With muscles exhibiting a strain-stiffening response, it is possible hydrostats could have developed entangled muscle fibers, but further anatomical investigation of the trunk structure is necessary. This challenge is met by roboticists and materials engineers working to understand how to mimic complex muscle structures through different means. Traditional forms of art and design have been an inspiration for finding new types of realistic muscle models, including origami, kirigami, and knitting.

Soft robotic designs require high degrees of freedom, but current actuators limit technology's ability to meet this goal. To mimic the iDOF of hydrostat motions, origami entwined actuation systems may be used to achieve these higher degrees of freedom. Shape-shifting structural design can create meta-materials that allow for both the elasticity and stability that soft robotic designs desire (Holmes, 2019). Origami, a traditional Japanese art form that uses folding paper techniques to create unique sculptures and topographies, can be coupled with bio-inspiration to create flexible but stable designs. Folding techniques, like Kresling origami, can provide mono-stability, bi-stability, and tri-stability, which then can be used to mimic nonlinear bio-mechanisms (Huang et al., 2022). This technique derives inspiration from skeletal joints but expansions to soft materials and soft robotics could be seen in the future.

While origami can be used as inspiration in structures and actuators, this method solely utilizes folding to achieve these various degrees of freedom and shapes. Kirigami is a variation of origami that instead uses both cutting and folding to create 3D and transformable structures. Previous research has incorporated kirigami to create stretchable materials with unique mechanical properties to achieve motion in soft robotics (Rafsanjani et al., 2018). Paired with bio-inspiration, kirigami has also been used to mimic different snake skins to improve friction and locomotion of robots and is still continuously used in bio-inspired soft robotic design as a flexible skin with directional friction (Branyan et al., 2020; Wei and Ghosh, 2022). To aid in the development of transformable kirigami structures, recent research also outlines new methods for developing and optimizing kirigami-inspired meta-materials, simplifying kirigami pattern design to elementary linear algebra (Dudte et al., 2022 preprint). Another way to integrate function with design is through knitting. The act of knitting involves manipulating 1D string-like material into a complex 2D lattice, allowing it to be an accessible technique to create and program complex meshes. This mesh manipulation impacts the overall mechanics and elastic nature of the fabric. This can lead to the application of knitting in bio-inspired materials (Singal et al., 2023 preprint) (Fig. 2). Researchers are working on programming these knits to work and move in various shapes, geometries, and structures (Si et al., 2023 preprint) (Fig. 2), including inspiration from the elephant skins' wrinkled and folded structure (Schulz et al., 2022a). Other recent studies have used machine based knitted to enhance pneumatic actuator movement and deformation (Luo et al., 2022). These structures can be manipulated to exhibit auxetic behavior and form new types of shapes and strains that stretch the limits of some of the more traditional materials that look to simulate muscle. Additionally, hydraulically amplified self-healing electrostatic (HASEL) robotics actuators are soft robotic artificial muscles designed for creating more life-like robotics (Yoder et al., 2022) (Fig. 2). The design of these actuators mimic the function and functionality of muscles and their flexible, adaptive capabilities to challenge the limitations of current robotic design. These new types of innovations to understand specific hydrostat and soft robotic structures are challenged by how to collect data and scientific evidence of their high degree of freedom movements. New resources have emerged to help scientists understand these complexities.

### New resources

Recently scientists have started utilizing more computationally intensive techniques to examine muscle physiology at smaller scales and with more precision. The use of Artificial Intelligence (AI) and Machine Learning (ML) to study animal movement has recently made these computational tasks more possible. The push for open-source hardware and software has made these techniques highly accessible, allowing engineers, physicists, and biologists to begin understanding iDOF hydrostat motions, enabling the creation of bio-inspired machines and robots. The advancement of soft robotics has developed in parallel with studies of hydrostats, as the primary function of soft robots is robots without joints, bones, or rigid structural material. These robots began as bio-mimetic structures, which only recreated the geometrical shape of the hydrostats that inspired them (Kim et al., 2013), but have evolved in recent years to include morphological and behavioral similarities such as suction-aided grasping and prehensile wrapping in the elephant (Schulz et al., 2021, 2023b).

As soft robotics grows in the scientific community, the functionality of materials and systems that can handle local deformations proves its benefits towards robotic technological advancement. Additive manufacturing methods provide many solutions to creating soft materials used in soft robotics. Additive manufacturing can create soft matter with different properties giving anisotropic behavior in all three dimensions (Truby and Lewis, 2016). These methods can be combined with soft robotics by utilizing various chemical and mechanical properties of polymers to allow these printed structures to have actuation potential (Wallin et al., 2018) and even actuation systems (Tyagi et al., 2021). Additive manufacturing is commonly associated with rigid matter such as traditional PLA printing. Still, the expansion of soft material and robotic printing gives way to new resources for mimicry of biological systems (Zhan et al., 2022) with future work potentially including hydrostat motion.

Additionally, computer vision and machine learning tools are advancing in species diversity to observe and track movement patterns and individual behavior for conservation biology applications and beyond (Tuia et al., 2022). DeepLabCut (DLC) is an example of an open-source toolkit that allows for the tracking of individual or many species of animals for neuromechanics and biomechanics alike. Recently DeepLabCut's innovations with ModelZoo and other open-sourced research tools have expanded the understanding of motion tracking for a wide range of species (Ye et al., 2023 preprint), allowing for analysis of animals as well as simulated robotic motion through markerless position tracking (Lauer et al., 2022; Kirkpatrick et al., 2022). The advanced computation techniques have also shown modeling advances with Soft Motion Gym (SoMoGym) (Graule et al., 2022) new toolkits for investigating soft robotic modeling and control. SoMoGym is an adaptable software toolkit that provides a benchmark for soft robotic training using reinforcement learning (RL), which could be an additional technique to create and adjust controls for soft robotic systems. Using open-source tech can allow for more robust solutions (Cornette et al., 2022) and there are datasets through programs such as WILDME where, by using computer vision and machine learning with crowdsourcing, you can find additional information on hydrostat movements and the development of different species which utilize these movements. Additionally, using human-based techniques to understand complexities in non-human skin and muscle has become more familiar with scientists using techniques such as second harmonic generation microscopy (Sadeghinia et al., 2022), which has been used to view individual collagen fiber orientation in the trunk skin of elephants (Sordilla et al., 2021) morphology and microstructure similar to the individual collagen orientation seen in human-skin (Boyle et al., 2019).

### New hypotheses

The most promising push in the science of hydrostats space is the testing of hydrostats and muscle understanding through novel hypotheses. The original definition of a hydrostat includes the assumption of conservation of volume; however, with the findings of asymmetry in many hydrostat systems including elephant trunk structure, the narrowness of this definition should be examined (Schulz et al., 2022b). With elephants having nostrils that increase in volume during fluid inhalation, how is the volume change displaced (Schulz et al., 2021)? With dorsal and ventral skin differences during elongation, are there any tasks that cause a volumetric change in a hydrostat as complex as the elephant trunk (Schulz et al., 2022b)? How do muscle arrangement and orientation directly govern a hydrostat's ability to move (Dagenais et al., 2021)? And what role does skin play in the hydrostat hypothesis, e.g. can skin or nostril dilation play a role in inhibiting muscle movement in hydrostats (Schulz et al., 2022b, 2023b)? Additionally, future studies should look into the role that internal connective tissue (or fascia) plays in hydrostat motion, as much of current studies focus on the dermal layer (Schulz et al., 2022b). These trade-offs between muscles and skin have encouraged soft roboticists to look at utilizing multiple structural levels in the bio-inspiration of hydrostats, not exclusively the muscular mechanism, to maintain function (e.g. skin wrapping). Additionally, how does the brain of these hydrostats discretize their movements? If hydrostats are partitioned into varying sections to allow for perception to take place, then are these partitioned sections under different movement constraints? These sections are differential and mapped directly to the brain in a similar system to that of vibrissae hairs (Kaufmann et al., 2022). Further study and understanding are needed to see how octopus, chameleons, and other morphologically specialist hydrostats control their appendages.

Hydrostats have a complex structural arrangement of several different muscle groups working for complex movements (Kier and Smith, 1985). How are each of these muscle groups arranged and how do other structures like skin, connective tissue, and more play a role? Additionally, are there possible interactions between each of these layers in a tangled arrangement for functional benefits? With the idea of hydrostat muscle and skin composition being entangled, can this entanglement hypothesis be expanded to not just increased mechanical strength but also increased functionality such as gripping (Becker et al., 2022)? How can we use programmable materials to enforce the impacts of entanglement, and does that allow for an understanding of new hydrostat involvement in entanglement in skin or muscle? As we establish the versatility of knitted mechanics and its potential to generate textiles of complex geometry and elasticity, we can explore its potential to enhance the design of not only skin mimics but actuated hydrostats. Through knitted materials, we get closer to capturing skin's morphological and composition attributes (Schulz et al., 2022a). Muscular hydrostats can also be a driver of development and behavior in animals. The structure of the tongue in animals can be used to elucidate feeding patterns and diets. The tongue has a basic arrangement of muscles that can vary depending on the species' feeding habits. For example, some reptiles show a more well-developed muscular layer to allow for the thrusting motion associated with prey capture (Schwenk, 2021; Ishaya et al., 2022). Morphogenesis studies historically examine development and behavior separately, but by combining the study of molecular and cellular processes with organismal-level muscular influences, behavior, and morphological changes can be better predicted across species. The physical forces driven by hydraulic systems, such as pressure and flow, can help initiate morphogenetic events, especially in marine invertebrates (Stokkermans et al., 2022).

Here, we expand the idea of a hydrostat through their structure-function-mechanism relationship structures that perform complex motion without rigid-body actuation systems. Research such as pulvinar-driven plants (Sleboda et al., 2022) allows further comparative studies between plant and animal fluid-actuated structures.

## Future prospects

As a scientific community, we are just beginning to understand the complexities and diversity of hydrostats. The field of soft robotics generally gains inspiration from different muscular hydrostat and hydroskeleton mechanisms, and the prospects of understanding how these hydrostats control their movement could significantly increase the understanding of how to create more effective bio-inspired robots based on their mechanisms. These mimics and robots often only incorporate muscular structures. Still, researchers are recently incorporating asymmetrical structures as shells around soft robots to give strength, flexibility, or dexterity (Sanchez et al., 2023). These outer skins of different knitted fabric types and materials can have dramatically different stress-strain responses allowing for bending and other manipulations (Sanchez et al., 2023). These asymmetries can lead to ideas of entanglement in these soft robots, allowing for activated entanglement allowing grasping of different topologies (Becker et al., 2022). Hydrostats have structures and functions at the micro-scale that can influence macro-level movements, including asymmetric skin geometry and mechanical structures (Schulz et al., 2022b, 2023b). In the future, researchers should be encouraged to look across scales at how these biological structures and arrangements at the micro level can influence macro-level behaviors, e.g. the interaction between skin-muscle-hair in mammals allowing for the expansion of bio-inspiration based solely on muscles.

As we look towards future studies of hydrostat systems, the classical models of muscular hydrostats mammalian tongues, elephant trunks, and octopus tentacles remain substantial. Yet, expanding model organisms beyond these classical hydrostats is essential for understanding more about the field. Considering the diversity of structure, functions, and mechanisms of hydrostats, we encourage future authors to think in the Inverse Krog Principle, which encourages that all organisms are worth studying (Clark et al., 2023). Scientists should study unique and novel hydrostats that have not been studied before, such as expanding our knowledge of plants and fungi in the hydrostats world. By expanding beyond these forms and functions of classical models, we can look to new types of movements. When studying specialized species that have evolved hydrostat-like structures, it is essential to remember that these structures were discovered because these animals still exist. We are seeing a rapid decline of our bio-diversity, and new ideas and techniques in fields such as conservation physiology can be used not only to study these species but also to gain valuable insight into how we can conserve those in decline (Jacobson et al., 2022; Plotnik and Jacobson, 2022; Tuia et al., 2022). Using new technology such as DeepLabCut has insights into the field of biomechanical understanding, as well as generating new types of conservation implications (Schulz et al., 2023a preprint) and by functioning jointly with zoological and aquarium organizations, interdisciplinary collaborations can open pathways for more informed research on hydrostat systems (Schulz et al., 2022c).
